# Investigation into Rheological Behavior of Warm-Mix Recycled Asphalt Binders with High Percentages of RAP Binder

**DOI:** 10.3390/ma16041599

**Published:** 2023-02-14

**Authors:** Hui Xu, Yiren Sun, Jingyun Chen, Jiyang Li, Bowen Yu, Guoqing Qiu, Yan Zhang, Bin Xu

**Affiliations:** 1 School of Transportation and Logistics, Dalian University of Technology, Dalian 116024, China; 2 City Institute, Dalian University of Technology, Dalian 116600, China; 3 Research and Development Center of Transport Industry of New Materials, Technologies Application for Highway Construction and Maintenance (Zhong Lu Gao Ke (Beijing) Road Technology Co., Ltd.), Ministry of Transport, Beijing 100088, China

**Keywords:** asphalt binder, warm-mix asphalt (WMA), reclaimed asphalt pavement (RAP), rheological behavior, performance

## Abstract

The rheological properties of warm-mix recycled asphalt binders are critical to enhancing design quality and interpreting the performance mechanisms of the corresponding mixtures. This study investigated the rheological behavior of warm-mix recycled asphalt binders with high percentages of RAP binder. The effects of two warm-mix additives [wax-based Sasobit (S) and surfactant-based Evotherm-M1 (E)], a rejuvenating aging [ZGSB (Z)], four RAP binder contents (0%, 30%, 50% and 70%), and three aging states (unaged, short-term aged and long-term aged) were evaluated in detail using the dynamic shear rheometer (DSR), bending beam rheometer (BBR) and Brookfield rotational viscometer tests as well as conventional performance tests over the whole range of temperatures. The results showed that the rejuvenating agent Z effectively alleviated the aging effect of the RAP binder; however, it could hardly eliminate entirely this negative impact, especially at higher RAP binder contents. The addition of S remarkably lowered the apparent viscosity of the warm-mix recycled binders by up to 35.0%, whereas E had little influence on the binder viscosity due to its surfactant nature. Besides, S performed much better in improving rutting resistance (with the increase of up to 411.3% in |G*|/sin*δ*) than E, while E exhibited superior fatigue performance (with the reduction of up to 42.3% in |G*|·sin*δ*) to that of S. In terms of the thermal cracking resistance, E had very slight influence and S even yielded an adverse impact (with the increase of up to 70.2% in *S*_a_ and the decrease of up to 34.1% in m-value). Further, S broadened the ranges of pavement service temperatures by about 12 °C, whereas E almost did not change the PG grades of the binders. Finally, regarding the characteristics of viscoelastic master curves, S considerably improved the dynamic modulus and lowered the phase angle of the binders over a wide range of frequencies and temperatures but led to the failure of the time-temperature superposition principle due to its thermorheologically complex nature. Nevertheless, in this regard, the effect of E was found very mild.

## 1. Introduction

The reuse of reclaimed asphalt pavement (RAP) in new asphalt mixtures is becoming increasingly prevalent in the asphalt paving industry [1,2,3]. RAP milled up or ripped off from old worn-out pavements contains valuable crushed aggregates and aged/stiff binder, and its recycling can conserve natural resources and save money, thus contributing to significant environmental and economic benefits. The aging of asphalt binder occurs owing to the loss of volatiles and oxidation during the whole process of production, construction, and service of asphalt mixture. The aged binder in RAP becomes significantly stiffer and, therefore, can furnish more desirable rutting resistance for the recycled asphalt mixture. Nevertheless, high RAP contents may cause a few typical problems, e.g., inferior fatigue cracking resistance, workability, and blending efficiency [4,5,6]. To eliminate these potential deficiencies, the percentages of RAP applied to the pavement surface layers are commonly limited below 25% by many highway agencies.

Over the past few years, applying warm-mix asphalt (WMA) technologies to RAP mixtures has attracted numerous interests from the asphalt pavement community [7,8,9,10,11,12]. Currently, three principal types of WMA technologies are involved, i.e., the chemical additives, organic additives, and foaming processes [13,14]. As low-carbon sustainable products/processes, WMA technologies enable asphalt mixtures to possess superior workability by reducing binder viscosity or raising lubrication. The mixing and compaction temperatures of WMA can thus be lowered by about 20~40 °C compared with those of the traditional hot-mix asphalt (HMA) [14]. This advantage not only helps decrease fuel consumption but also curbs the emissions of asphalt fumes and greenhouse gas in construction. Due to the lower degree of asphalt aging caused by reduced temperatures in production and compaction, WMA technologies tend to provide better fatigue performance but poorer rutting resistance for asphalt mixtures [14]. Taking into account these factors, the combined utilization of the WMA and RAP can rationally avoid the drawbacks of both technologies and effectively improve the percentages of RAP in the recycled mixtures.

Some research attempts have been made to investigate the performance of warm-mix recycled asphalt mixtures with high RAP contents. Zhao et al. [7] comparatively assessed the joint influence of two WMA technologies (foaming and Evotherm) and high RAP contents (up to 40%) on the rutting, moisture, and fatigue susceptibility of 15 mixes and found that rutting might be a potential issue for WMA-high RAP mixtures. Mogawer et al. [15] evaluated the performance of asphalt rubber surface mixtures with RAP percentages up to 40% and a wax-based WMA additive. They found that high-content RAP had an adverse effect on the fatigue and reflective cracking resistance of the mixtures; however, the WMA technology could mitigate this impact. Xiao et al. [16] analyzed Superpave mix design characteristics of various high percentages (up to 50%) of RAP in terms of two WMA technologies (Evotherm additive and foaming technology). Song et al. [17] compared the influence of a rejuvenating agent and a foaming technology commonly used in the United States on the performance of asphalt mixtures containing 50% RAP and observed that both technologies improved the moisture susceptibility, but they exhibited opposite effects on rutting performance. Wang et al. [18] assessed multiple performances, including moisture susceptibility, low-temperature, high-temperature, and fatigue, of WMA recycled mixtures with two RAP contents (50% and 70%), two WMA additives (wax R and surfactant M) and two gradations (AC-16 and AC-13).

Another important issue lies in evaluating the rheological properties of warm-mix recycled asphalt binders, which can facilitate enhancing the design quality and interpreting the performance mechanisms of the corresponding mixtures. Xiao et al. [19] analyzed the rutting and fatigue performance of asphalt binders with high contents (up to 50%) of RAP binders and a warm-mix additive. Yu et al. [20] evaluated the rheological properties of foamed warm-mix recycled asphalts with different RAP binder contents (up to 80%) using the Brookfield rotational viscometer and dynamic shear rheometer (DSR) at both high- and intermediate-temperatures. Sun et al. [21] assessed the impacts of two WMA additives on the rutting and fatigue performance of binders with high contents (30~70%) of RAP binder by the multiple stress creep and recovery (MSCR) and linear amplitude sweep (LAS) tests.

Despite these studies involving this topic, they were mostly concentrated on the rheological properties over only part of the service temperature range (e.g., high- or intermediate-temperature). Besides, very limited research efforts have been devoted to exploring the combined influence of the rejuvenating agent, WAM additives/processes, and high-content RAP binder on the rheological properties of warm-mix recycled asphalt binders. To this end, this study investigated the rheological behavior of warm-mix recycled asphalt binders with high percentages of RAP binder over the entire service temperature range (low-, intermediate-, and high-temperature). The influence of a rejuvenating agent, two warm-mix additives, four RAP binder contents (up to 70%), and three aging states (unaged, short-term aged, and long-term aged) were evaluated.

## 2. Materials

A #90 asphalt (penetration grade 80/100) was selected as the virgin binder. An actual RAP binder was considered in this study, which was extracted and recovered from a RAP collected from a highway in Dalian, China. To have a grasp of the two binders, their basic properties were measured, as displayed in Table 1. It can be seen that the actual RAP binder exhibited inferior low- and intermediate-temperature performance but superior high-temperature performance to that of the virgin binder (#90).

Given that laboratory-aged asphalts always display relatively more reproducible and stable rheological behaviors, an artificial RAP binder was produced as an alternative to the actual RAP binder for the purpose of this study, which was fabricated by subjecting the #90 asphalt to successive rolling thin-film oven (RTFO) aging [22] and pressurized aging vessel (PAV) aging [23]. The short-term RTFO aging was performed at 163 °C for 85 min, and the long-term PAV aging was performed at 100 °C. To ensure similar performance and aging degree of the artificial RAP binder to the actual one, three different exposure durations in the PAV, 10, 15, and 20 h, were applied, and accordingly, the artificial RAP binders were denoted as PAV-10, PAV-15, and PAV-20, respectively. In this study, four RAP binder percentages, 0%, 30%, 50%, and 70%, were adopted to analyze the effect of high RAP binder contents.

**Figure 1:**
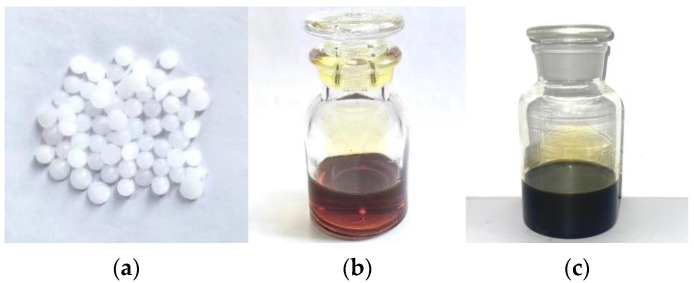
Images of the WMA additives and rejuvenating agent: (**a**) Sasobit; (**b**) Evotherm-M1; (**c**) ZGSB.

To investigate the influence of WMA additives, two different WMA additives, Sasobit and Evotherm-M1, were employed. Sasobit is a white organic synthetic hard wax produced from coal gasification through the Fischer-Tropsch (FT) method, which can reduce the binder viscosity and thus decrease the production and placement temperatures. Sasobit is described as an “asphalt flow improver”, and its use enables production temperatures to be reduced by 20~30 °C [24]. Evotherm-M1 is an amber chemical liquid surfactant that can enhance the coating of asphalt binder to aggregates and thus improve the workability of asphalt concrete. The Evotherm-M1 belongs to the Evotherm 3G product, which is a water-free warm-mix technology [14]. The dosages of 3% and 0.5% by weight of the total binder were used for Sasobit and Evotherm-M1, respectively, according to the suppliers’ recommendations. Figure 1 shows the images of the two WMA additives.

Besides, a rejuvenating agent ZGSB (Figure 1), was used to restore the performance of the RAP binder. Table 2 presents the basic properties of ZGSB. To determine the optimal content of ZGSB, four dosages, 2%, 4%, 6%, and 8% by weight of the RAP binder, were taken into account.

For brevity, the two WMA additives, Sasobit and Evotherm-M1, and the rejuvenating agent, ZGSB, are respectively designated as S, E, and Z in the following analysis. Thus, three categories of recycled binders are involved, in which the Z binders represent those containing only the rejuvenator ZGSB, the S + Z binders represent those containing both Sasobit and ZGSB and the E + Z binders represent those containing both Evotherm-M1 and ZGSB.

## 3. Methodology

### 3.1. Conventional Performance Tests

According to the standard test methods of China JTG E20-2011 [25], three conventional performance tests, i.e., the penetration, softening point, and ductility tests, were conducted to evaluate the basic performance of the binders.

### 3.2. Apparent Viscosity Test by Brookfield Rotational Viscometer

The apparent viscosity was measured at four temperatures, 115, 135, 155, and 175 °C, in accordance with ASTM D4402 [26] using a Brookfield rotational viscometer. The apparent viscosity can well reflect the workability of the binders during production and construction.

### 3.3. High- and Intermediate-Temperature Performance Test

The high- and intermediate-temperature performance of asphalt binders can be characterized using a DSR (Lab^+^, Malvern) according to ASTM D7175 [27]. In this method, the dynamic shear modulus |G*| and phase angle *δ* at the loading frequency of 10 rad/s are obtained.

Further, the high-temperature rutting performance can be assessed using the rutting parameter |G*|/sinδ, which is derived from the dissipated energy density per oscillatory loading cycle *W*_d_ at a constant stress amplitude *τ*_a_:

(1) $$W_{d}=\pi {\left(\tau_{a}\right)}^{2}/\left(\left|G^{*}\right|/\sin\delta \right)$$

In the rutting performance testing, the binders under both the unaged original state and the short-term RTFO aged state are required.

Similarly, the intermediate-temperature fatigue performance can be evaluated using the fatigue parameter |G*|·sin*δ*, which is calculated from the dissipated energy density per oscillatory loading cycle *W*_d_ at a constant strain amplitude *γ*_a_:

(2) $$W_{d}=\pi {\left(\gamma_{a}\right)}^{2}\left(\left|G^{*}\right|\cdot \sin\delta \right)$$

In the fatigue performance testing, the binder subjected to successive short-term RTFO aging and long-term PAV aging is required.

Two types of plates were adopted in testing. The 8-mm plates with a gap of 2 mm were used at intermediate temperatures for evaluating the fatigue performance, while the 25-mm plates with a gap of 1 mm were employed at high temperatures for evaluating the rutting performance.

### 3.4. Low-Temperature Performance Test

The thermal cracking resistance of asphalt binders at low temperatures can be assessed using a BBR (Cannon Instrument) in accordance with ASTM D6648 [28]. In this method, the flexural-creep stiffness *S*_a_(*t*), creep compliance *D*_a_(*t*) = 1/*S*_a_(*t*) and m-value of asphalt binder are determined through a creep process. The fixed contact load *P* around 980 mN is applied to the mid-point of the prismatic test specimen for 240 s, and the mid-span deflection *ξ*(*t*) was monitored at an interval of 0.5 s. The flexural-creep stiffness *S*_a_(*t*) and compliance *D*_a_(*t*) can be calculated by the following:

(3) $$S_{a}(t)=\frac{1}{D_{a}(t)}=\frac{Pl^{3}}{4bh^{3}\xi (t)}$$

where *l* is the span length between the two supports; *h* is the thickness of the prismatic specimen; *b* is the width of the prismatic specimen; *t* is the loading time. Moreover, the m-value can be determined using the slope of the stiffness versus the time on the log-log scale. In the low-temperature performance testing, the binder undergoing both RTFO and PAV aging is required. In this study, the temperatures of −12, −18, and −24 °C were used for testing.

### 3.5. Frequency Sweep Test

To further evaluate the linear viscoelastic behavior over a wide range of frequencies and temperatures, the frequency sweep test was performed on the binders utilizing the DSR. The tests were carried out in a strain-controlled mode. At intermediate temperatures, the 8-mm plates with a gap of 2 mm were applied, and the angular strain amplitudes were set to 0.5%, while at high temperatures, the 25-mm plates with a gap of 1 mm were applied, and the angular strain amplitudes were set to 1%. The testing was conducted at −2, 4, 16, 28, 40, 52, and 64 °C, and at each temperature, the complex modulus G* was measured at the frequencies of 0.1~100 rad/s as follows [29]:

(4) $$G^{*}=G^{\prime }+i\cdot G^{\prime \prime },\;G^{\prime }=\left|G^{*}\right|\cos\delta ,\;G^{\prime \prime }=\left|G^{*}\right|\sin\delta$$

where $i=\sqrt{-1}$ is the imaginary unit; *G*′ is the storage modulus; *G*″ is the loss modulus.

With the measurements of G* obtained at different temperatures and frequencies, the corresponding master curves can be constructed following the time-temperature superposition principle. The time-temperature shift factor (*α*_T_) is represented on the frequency domain by the following [30]:

(5) $$\alpha_{T}=\frac{\omega_{r}}{\omega }$$

where *ω* is the angular frequency; *ω*_r_ is the reduced angular frequency.

The master curves of G* for asphalt binders can be modeled by the 2S2P1D model [31]. The 2S2P1D model is a complex-valued model consisting of two spring elements, two parabolic elements, and a dashpot element; thus, it can analytically represent the dynamic modulus and phase angle components as well as the storage modulus and loss modulus components. Besides, its continuous relaxation and retardation spectra can also be analytically derived [32]. The 2S2P1D model has the following form:

(6) $$G^{*}\left(\omega_{r}\right)=G_{e}+\frac{G_{g}-G_{e}}{1+\alpha {\left(i\omega_{r}\tau_{0}\right)}^{-k}+{\left(i\omega_{r}\tau_{0}\right)}^{-h}+{\left(i\omega_{r}\beta \tau_{0}\right)}^{-1}}$$

where *G*_e_ is the equilibrium modulus; *G*_g_ is the instantaneous modulus; *α*, *k*, *h*, *β,* and *τ*_0_ are the model parameters. Following a common practice, *G*_e_ and *G*_g_ were set to 0 Pa and 10^9^ Pa in this study.

In the development of the master curves, the parameters of the 2S2P1D model and the time-temperature shift factors can be calculated simultaneously. The target error function to minimize, *f*, is given by the following:

(7) $$f=\frac{1}{N}\sqrt{{\sum_{i=1}^{N}\left(\frac{{\left|G^{*}\right|}_{\mathrm{mea},i}-{\left|G^{*}\right|}_{\mathrm{cal},i}}{{\left|G^{*}\right|}_{\mathrm{mea},i}}\right)}^{2}}+\frac{1}{N}\sqrt{{\sum_{i=1}^{N}\left(\frac{\delta_{\mathrm{mea},i}-\delta_{\mathrm{cal},i}}{\delta_{\mathrm{mea},i}}\right)}^{2}}$$

where *N* is the number of the data points; the subscripts mea and cal refer to measured and calculated values, respectively.

### 3.6. Experimental Procedure

**Figure 2:**
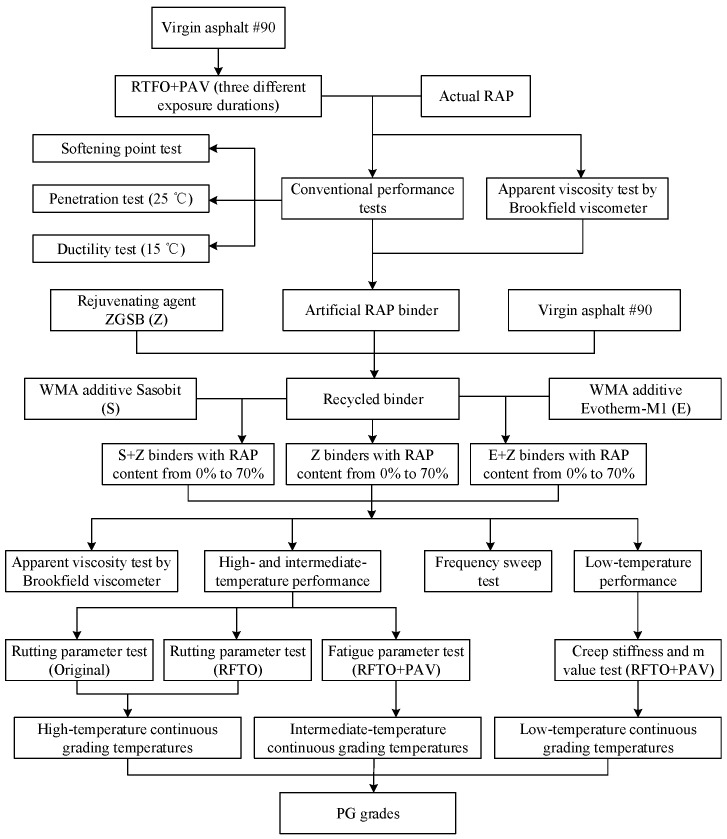
Flowchart of the experimental procedure.

Figure 2 shows the experimental procedure of this study. First, an artificial RAP binder was fabricated as an alternative to the actual RAP binder. Then, the rejuvenating agent content was determined to generate the recycled binders with different RAP binder contents. Further, the two WMA additives were added to produce the warm-mix recycled binders. Finally, a variety of rheological tests were carried out to analyze the rheological behavior and determine the continuous grading temperatures and PG grades of the binders.

## 4. Results and Discussion

### 4.1. Determination of PAV Aging Time for Artificial RAP Binder

**Figure 3:**
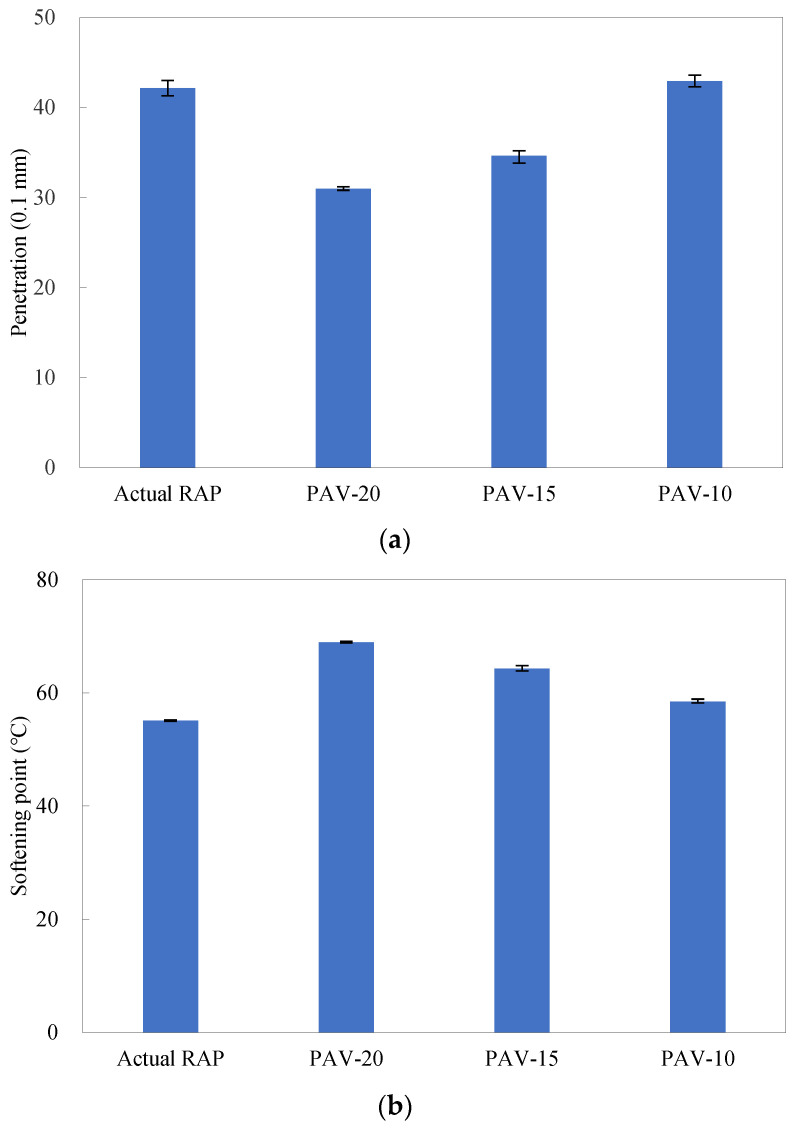

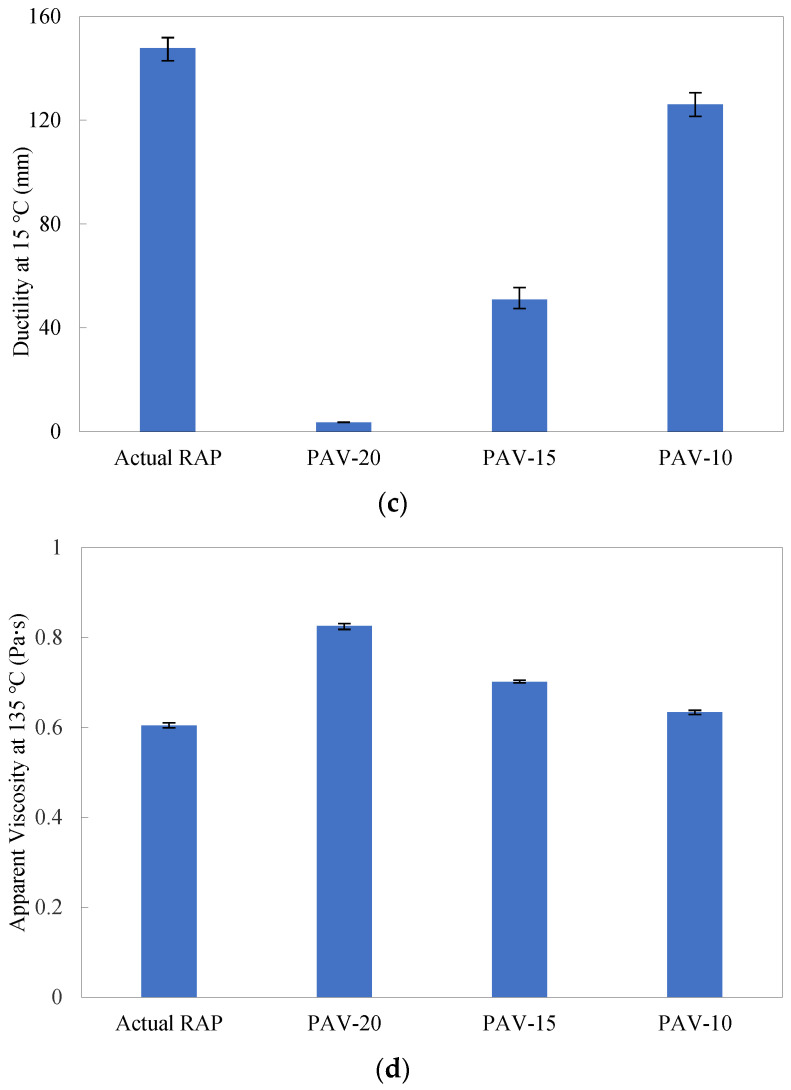
Basic properties of artificial RAP binders aged at different PAV exposure times: (**a**) penetration; (**b**) softening point; (**c**) ductility; (**d**) apparent viscosity.

As mentioned above, three PAV exposure times, 10, 15, and 20 h, were applied to manufacturing the artificial RAP binder to achieve a similar performance and aging degree to those of the actual one. Figure 3 presents the basic properties of the artificial RAP binders aged at different PAV exposure times. As can be seen, the penetration and ductility (15 °C) reduced, and the softening point and apparent viscosity rose with the increasing exposure time, indicating that the artificial RAP binder became increasingly stiffer. The artificial RAP binder aged for 10 h, PAV-10, exhibited very similar basic properties to those of the actual RAP binder; thus, the RAP-10 binder was used as an alternative to the actual RAP binder in this study.

### 4.2. Determination of Rejuvenating Agent Content

**Figure 4:**
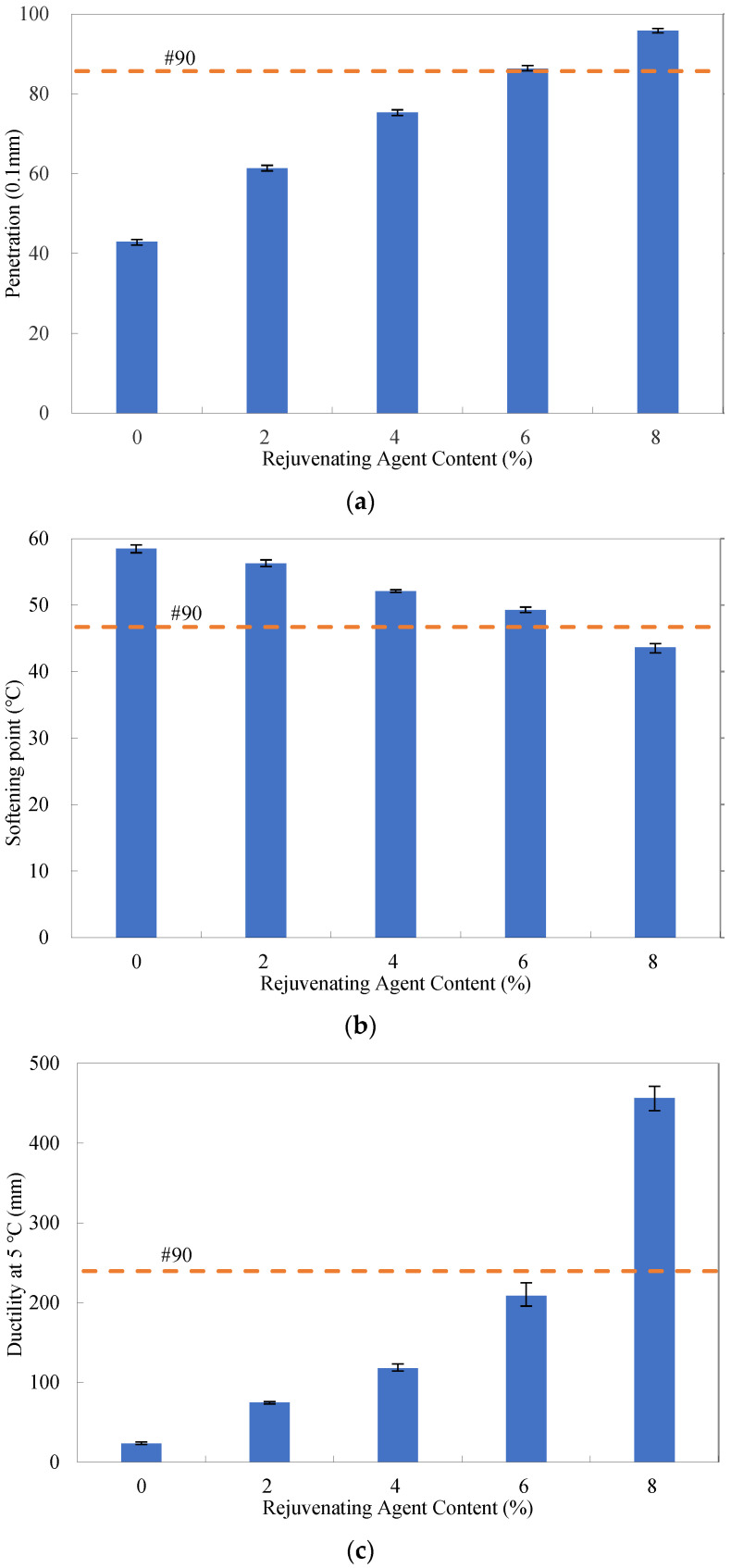

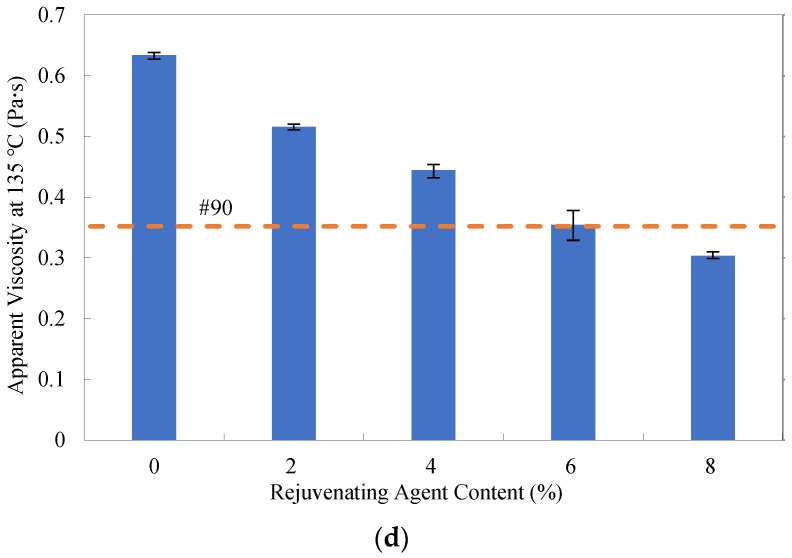
Basic properties of rejuvenated asphalt binders at different rejuvenating agent contents: (**a**) penetration; (**b**) softening point; (**c**) ductility; (**d**) apparent viscosity.

To recycle the RAP binder, the rejuvenating agent content needs to be appropriately determined. In this study, the basic properties of the binders, including the penetration, softening point, ductility, and apparent viscosity, were used to this end, as shown in Figure 4. It should be mentioned that since the ductility values of all the binders were larger than 100 cm at 15 °C, the ductility tests were conducted at 5 °C to more clearly reveal the effect of the rejuvenating agent content herein. It can be observed that as the rejuvenating agent content increased, the recycled binder became softer significantly. At the content of 6%, the properties of the recycled binder were found most close to those of the original #90 binder; therefore, the rejuvenating agent dosage of 6% by weight of the RAP binder was employed in the present study.

### 4.3. Analysis of Apparent Viscosity Test Results

**Figure 5:**
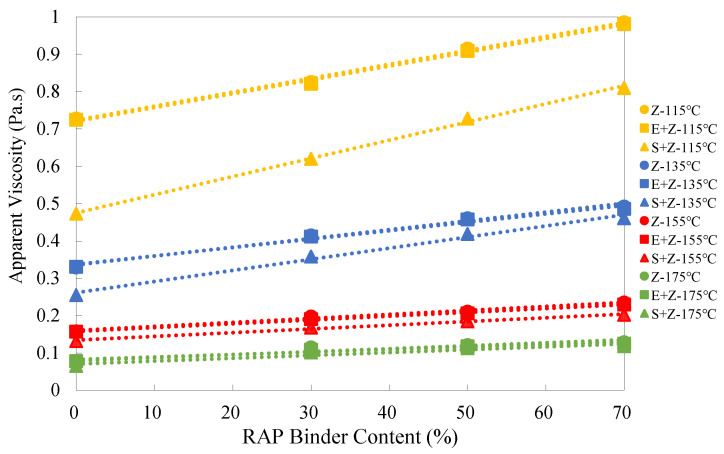
Apparent viscosity test results of the warm-mix recycled binders at different RAP binder contents and testing temperatures.

Figure 5 shows the apparent viscosity test results of the warm-mix recycled binders at different RAP binder contents and testing temperatures. As observed, the apparent viscosity values of all three types of recycled binders (i.e., Z, S + Z, and E + Z) increased linearly with the RAP binder content. This indicates that although a relatively accurate rejuvenating agent dosage by weight of the RAP binder (6%) was used, it was still difficult to completely offset the aging effect, especially for higher RAP binder contents.

It can be seen from Table 3 that the S + Z binders exhibited the highest percentage increase of apparent viscosity, e.g., 71.2% and 81.5%, respectively, at 115 °C and 175 °C for the RAP binder content of 70%, whereas the E + Z binders presented the lowest percentage increase of apparent viscosity, e.g., only 35.3% and 60.3%, respectively, at 115 °C and 175 °C for the RAP binder content of 70%. This indicates that the WMA additive S can raise the RAP binder content susceptibility of apparent viscosity while E has the opposite effect.

Moreover, Figure 5 shows that at the four different temperatures, the viscosity values of the S + Z binders were lower than those of the E + Z and Z binders, and as the temperature decreased, the viscosity differences between the S + Z binders and the other two types of binders became more remarkable. This indicates that the WMA additive S plays a critical role in reducing the binder viscosity. Thus, it allows a higher RAP binder content. On the other hand, the viscosity values of the E + Z and Z binders were very close to each other, and this indicates that the WMA additive E has little influence on the binder viscosity. Clearly, the WMA additive E operates by a mechanism different from that of S. Actually, as a surfactant, E affects the chemical bonding between binder and aggregate rather than the binder viscosity. 

Table 4 shows that the addition of S could decrease the apparent viscosity by up to 35.0% and 12.1%, whereas the use of E could only decrease the apparent viscosity by up to 0.7% and 5.2%, respectively, at the temperatures of 115 °C and 175 °C.

### 4.4. Analysis of High-Temperature Performance Test Results

**Figure 6:**
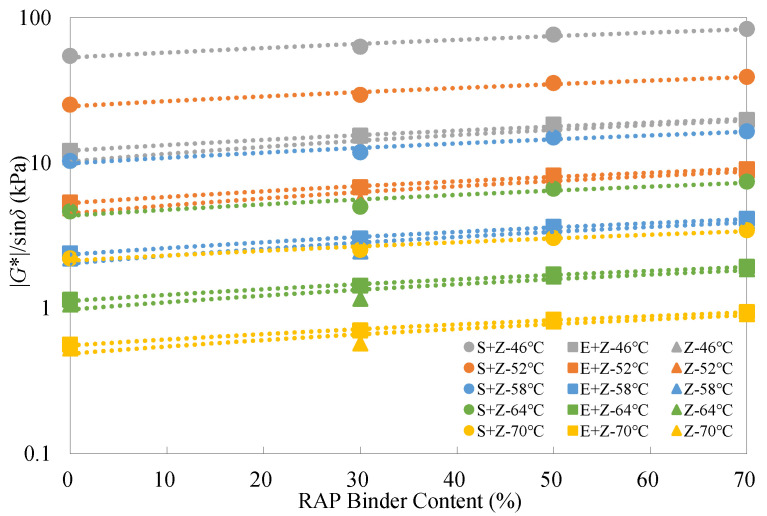
Rutting parameter test results of the warm-mix recycled binders at different RAP binder contents under the unaged condition.

Typically, a higher |G*|/sin*δ* value represents a superior rutting resistance. Figure 6 presents the rutting parameter test results of the warm-mix recycled binders at different RAP binder contents under the unaged condition. As can be seen, the rutting parameter values exhibited linear increasing trends with the RAP binder content on the logarithmic scale regardless of WMA additives used, indicating an improvement in rutting performance. It is seen that the difference of |G*|/sin*δ* between the binders with and without the RAP binder increased considerably with the rising RAP binder content. Thus, accurately controlling the rejuvenating agent content is critical to guaranteeing the performance of recycled asphalt mixtures with high RAP contents.

Table 5 shows that S could better alleviate the increase in |G*|/sin*δ* with the RAP binder content than E under the unaged condition. For instance, at the RAP binder content of 70% and 70 °C, the percentage increase of |G*|/sin*δ* for the S + Z binder was 56.2% while that for the E + Z binder was 67.5%, both of which were less than 72.3% for the Z binder.

Besides, Figure 6 presents that at any given RAP content and temperature, the S + Z binders displayed the best performance, followed by the E + Z binders, and the Z binders ranked the lowest. Although both WMA additives contributed to the increasing |G*|/sin*δ*, S performed significantly better than E. This may be because wax crystallization in the additive S reinforces the permanent deformation resistance of asphalt binders at elevated temperatures that are lower than the melting point of S (about 100 °C). 

Table 6 shows that the inclusion of S could increase |G*|/sin*δ* by up to 399.5% and 340.8%, but the use of E could only increase |G*|/sin*δ* by up to 20.6% and 23.0%, respectively, at the temperatures of 46 °C and 70 °C under the unaged condition.

**Figure 7:**
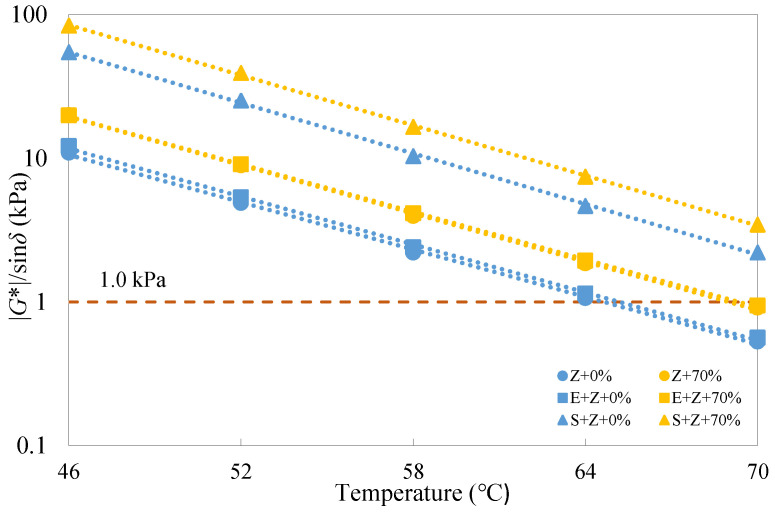
Rutting parameter test results of the warm-mix recycled binders at different testing temperatures under the unaged condition.

According to the Superpave PG specification ASTM D6373 [33], unaged binders should satisfy the requirement that |G*|/sin*δ* values are larger than 1 kPa. It can be observed from Figure 7 that the rutting parameter test results of the warm-mix recycled binders under the unaged condition were all greater than 1 kPa at testing temperatures below 64 °C; however, when the temperature rose to 70 °C, only the S + Z binders met the requirement, implying a more desirable rutting resistance for the S + Z binders than the E + Z and Z ones. The role of E in enhancing rutting performance was found to be quite slight at different temperatures, and this can be ascribed to its nature as a chemical surfactant.

**Figure 8:**
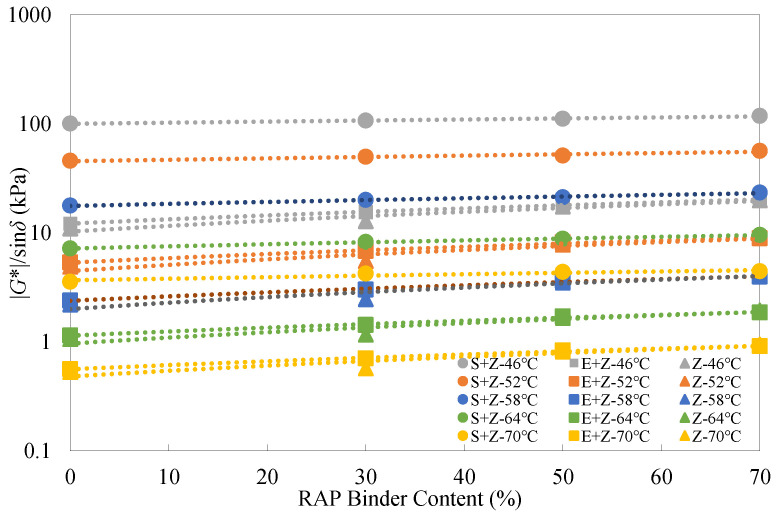
Rutting parameter test results of the warm-mix recycled binders at different RAP binder contents under the short-term aged condition.

**Figure 9:**
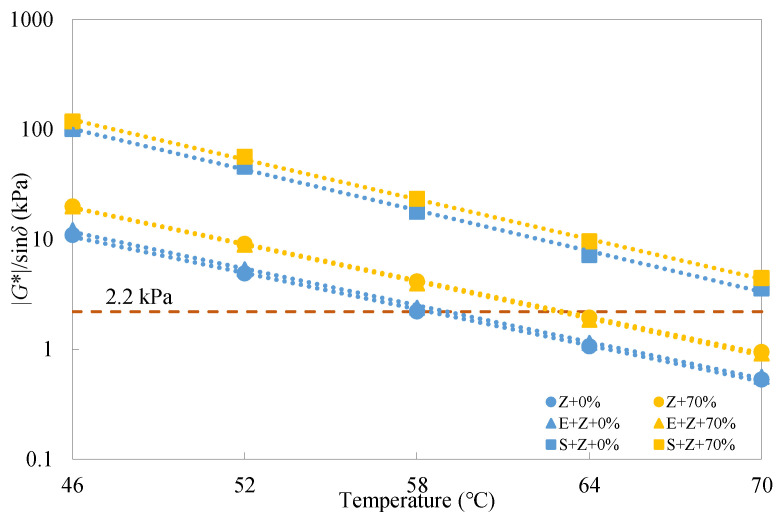
Rutting parameter test results of the warm-mix recycled binders at different testing temperatures under the short-term aged condition.

Figure 8 and Figure 9 give the rutting parameter test results of the warm-mix recycled binders at different RAP binder contents and testing temperatures under the short-term RTFO aged condition. In this case, the |G*|/sin*δ* values should be larger than 2.2 kPa. It is evident that similar observations to those under the unaged condition can be made for all the recycled binders under the short-term aged condition.

Table 7 shows that S could alleviate the increase in |G*|/sin*δ* with the RAP binder content, but E intensified this percentage increase under the short-term aged condition. This observation was different from that obtained under the unaged condition.

Table 8 indicates that the inclusion of S could increase |G*|/sin*δ* by up to 411.3% and 335.3%, but the use of E could only increase |G*|/sin*δ* by up to 29.9% and 26.0%, respectively, at the temperatures of 46 °C and 70 °C under the short-term aged condition.

### 4.5. Analysis of Intermediate-Temperature Performance Test Results

**Figure 10:**
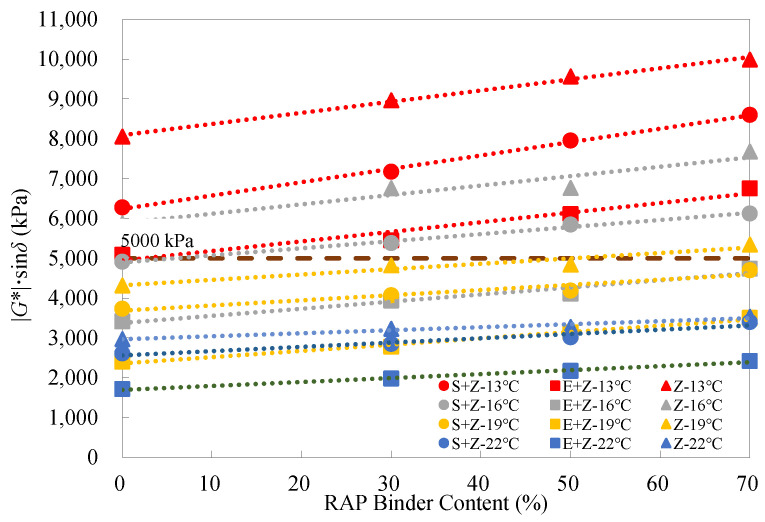
Fatigue parameter test results of the warm-mix recycled binders at different RAP binder contents and testing temperatures under the long-term aged condition.

Generally, a higher fatigue parameter |G*|·sin*δ* represents an inferior fatigue performance. Figure 10 displays the fatigue parameter test results of the warm-mix recycled binders at different RAP binder contents under the long-term aged condition. As the RAP binder content increased, the |G*|·sin*δ* values for all the binders increased linearly, suggesting that the RAP binder has an adverse impact on the fatigue resistance. Moreover, it is noticed that the rejuvenating agent was unable to entirely compensate for the negative effect of the aged binder despite a relatively accurate determination of the rejuvenating agent dosage. In addition, both the WMA additives S and E lowered the |G*|·sin*δ* values, which indicates that both S and E are capable of improving the fatigue performance of the recycled binders. Specifically, E performed better than S in this aspect.

Table 9 shows that both S and E could promote the increase in |G*|·sin*δ* with the RAP binder content, and the two WMA additives seemed to exhibit a comparable effect. Table 10 indicates that the addition of S could reduce |G*|·sin*δ* by up to 22.1%, and 12.1% and the use of E could even reduce |G*|·sin*δ* by up to 39.3% and 42.3%, respectively, at the temperatures of 13 °C and 22 °C under the long-term aged condition.

**Figure 11:**
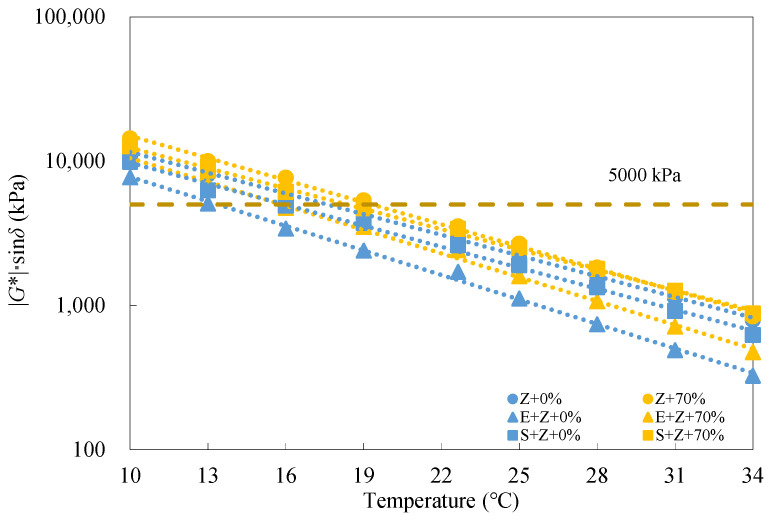
Fatigue parameter test results of the warm-mix recycled binders at different testing temperatures under the long-term aged condition.

In accordance with the Superpave PG specification ASTM D6373 [33], long-term aged binders should meet the requirement that the |G*|·sin*δ* values must be less than 5000 kPa. Figure 11 gives the |G*|·sin*δ* test results of the binders at different testing temperatures. At temperatures above 22 °C, the |G*|·sin*δ* values of all the binders could satisfy the criterion. At 19 °C, only the Z binders with the RAP content of 70% (Z + 70%) did not meet the requirement, whereas, at 16 °C, only the E + Z binders were desirable. When the temperature dropped below 13 °C, all the test results exceeded the limiting value (5000 kPa). It can be observed from the slopes of the fitted lines that in terms of fatigue resistance, the addition of E slightly elevated the temperature susceptibility of the recycled binders, but the inclusion of S had a very limited effect on the temperature susceptibility.

### 4.6. Analysis of Low-Temperature Performance Test Results

**Figure 12:**
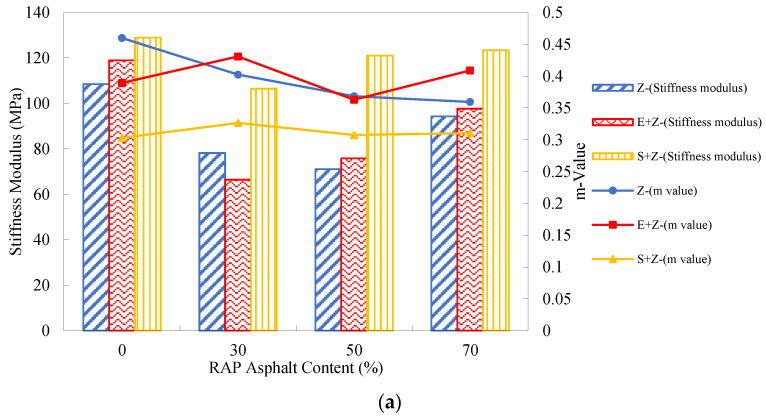

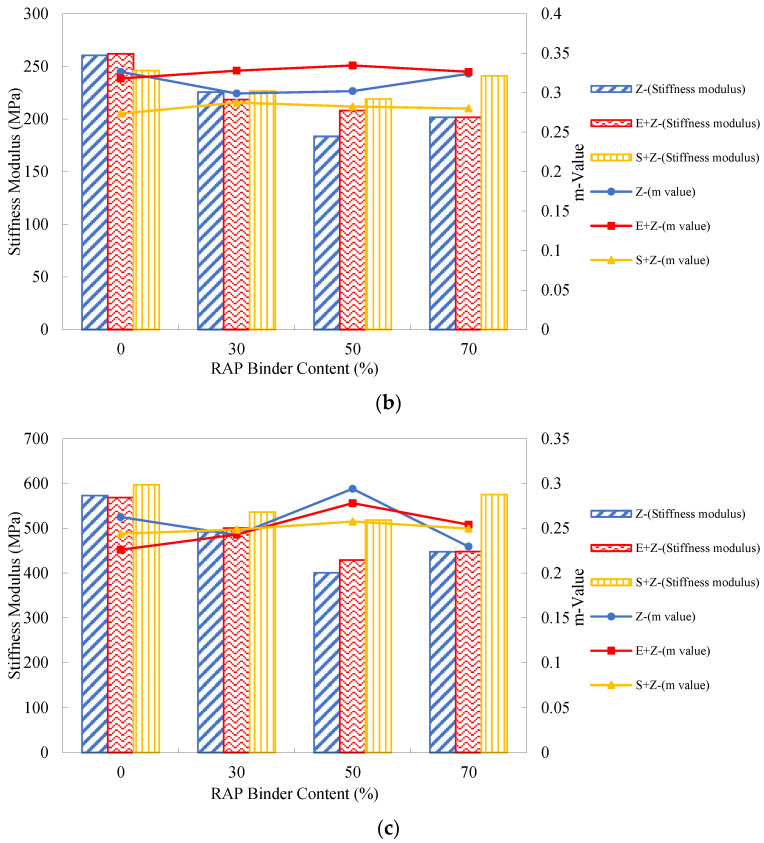
Creep stiffness and m value test results of the warm-mix recycled binders at different RAP binder contents and testing temperatures under the long-term aged condition: (**a**) −12 °C; (**b**) −18 °C; (**c**) −24 °C.

In general, the binder with a lower stiffness modulus *S*_a_ and a higher m-value has a more satisfactory low-temperature thermal cracking resistance. Figure 12 presents the creep stiffness and m-value test results of the warm-mix recycled binders at different RAP binder contents and testing temperatures. As can be seen, compared to the stiffness moduli at the RAP binder content of 0%, those at 30% to 70% were even lower at the three temperatures. This abnormal observation can actually be attributed to the addition of the rejuvenating agent Z, which has a softening effect on the aged binder. Additionally, with the growth of the RAP binder content, the m-values of the Z binders did not exhibit noticeable change, and this also demonstrates the effect of the rejuvenating agent. Table 11 shows no remarkable trends for the percentage increases of *S*_a_ and m-value with the RAP binder content.

Further, Figure 12 shows that regardless of the RAP percentage, the stiffness modulus values of the S + Z binders were all higher than those of the Z and E + Z binders, and the m-values of the S + Z binders were mostly lower than those of the other two types of binders. This implies that the use of S has a negative impact on the thermal cracking resistance. For the E + Z and Z binders, both stiffness moduli and m-values were very close to each other; thus, E has a very mild influence on the low-temperature rheological properties of the recycled binders. Table 12 presents that the addition of S could increase the *S*_a_ by up to 29.3%, 19.6%, and 70.2% and reduce the m-values by up to 12.4%, 16.2%, and 34.1%, respectively, at the temperatures of −24 °C, −18 °C, and −12 °C under the long-term aged condition.

In terms of the Superpave PG specification ASTM D6373 [33], the stiffness moduli of the long-term aged binders should be less than 300 MPa, but the m-values should be greater than 0.3. Obviously, at −12 °C, the stiffness moduli and m-values of all the binders were acceptable, but at −24 °C, were ineligible. At −18 °C, due to the limitation of the m-value, the Z + S binders at all the RAP binder contents and the Z binders at the RAP binder content of 30% were undesirable for thermal cracking resistance.

### 4.7. Analysis of Continuous Grading Temperatures and PG Grades

**Figure 13:**
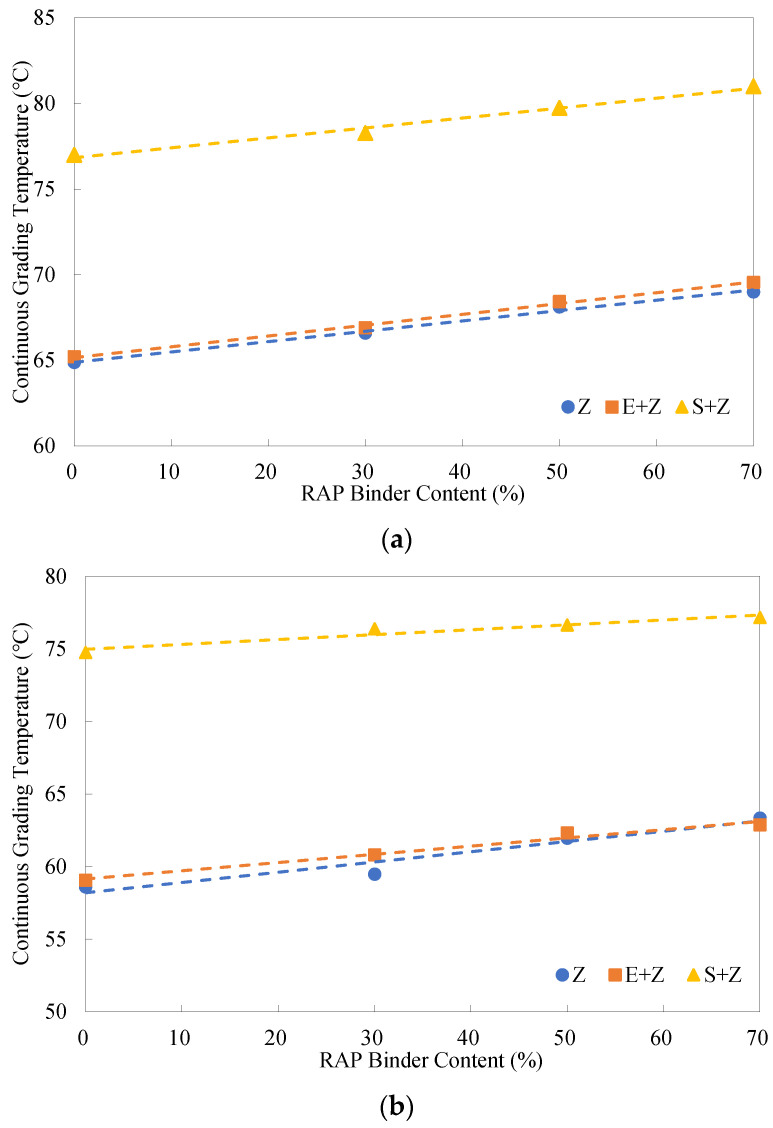
High-temperature continuous grading temperatures of the warm-mix recycled binders at different RAP binder contents: (**a**) original; (**b**) RTFO-aged.

In terms of the specification ASTM D7643 [34], the continuous grading temperatures of asphalt binder can be calculated by interpolating between test data measured at two adjacent specification temperatures so that high-, intermediate- and low-temperature grades can be more accurately evaluated. Figure 13 displays the resulting high-temperature continuous grading temperatures of the binders at different RAP binder contents. It is seen that as the RAP binder percentage rose, the continuous grading temperatures merely increased moderately (by about 4 °C from 0% to 70%) for both the original and RTFO-aged binders. This is because the addition of the rejuvenating agent (6% by weight of the RAP binder) alleviated the hardening effect of the aged binder to a certain extent. Compared to the continuous grading temperatures of the Z binders, the results for the S + Z binders increased by more than two high-temperature grades (12 °C), while the results for the E + Z binders solely increased slightly. This demonstrates the advantage of S over E in enhancing the rutting resistance.

**Figure 14:**
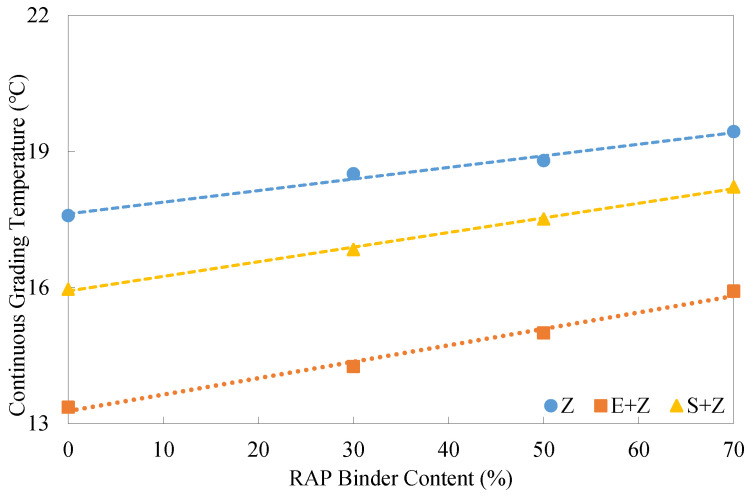
Intermediate-temperature continuous grading temperatures of the warm-mix recycled binders at different RAP binder contents.

Figure 14 gives the intermediate-temperature continuous grading temperatures of the warm-mix recycled binders at different RAP binder contents. The continuous grading temperatures of the three types of binders presented linear trends with the RAP binder content. The addition of E led to the greatest reduction of the continuous grading temperatures (more than a grade for fatigue performance, 3 °C), followed by the use of S.

**Figure 15:**
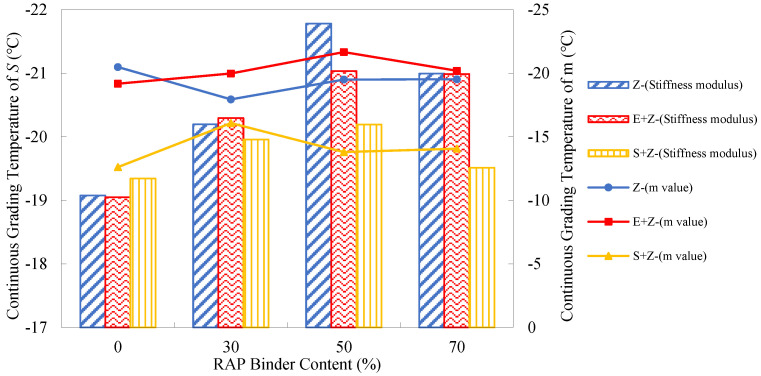
Low-temperature continuous grading temperatures of the warm-mix recycled binders at different RAP binder contents.

Figure 15 shows the low-temperature continuous grading temperatures determined using the stiffness modulus *S*_a_ and m-value, respectively. The final low-temperature grade is dependent on the upper of the two continuous grading temperatures from *S*_a_ and the m-value. Evidently, the inclusion of S considerably elevated the continuous grades, thus having a negative impact on the thermal cracking resistance. Unlike the WMA additive S, E almost did not change the low-temperature continuous grading temperatures of the Z binders.

Table 13 shows the resulting PG grades of all the warm-mix recycled binders according to the specification ASTM D6373 [33]. As can be seen, when subjected to short-term aging, the high-temperature grades of both the E + Z and Z binders declined by one grade (6 °C), whereas the S + Z binders mostly maintained the original grades, which implies the role of the WMA additive S in aging resistance. Additionally, the S + Z binders possessed wider ranges of pavement service temperature (wider by about two PG grades, 12 °C) than those of the other two types of binders though S lowered the low-temperature performance of the binders. In this regard, the use of E almost did not change the PG grades of the binders.

### 4.8. Analysis of Linear Viscoelastic Master Curves

**Figure 16:**
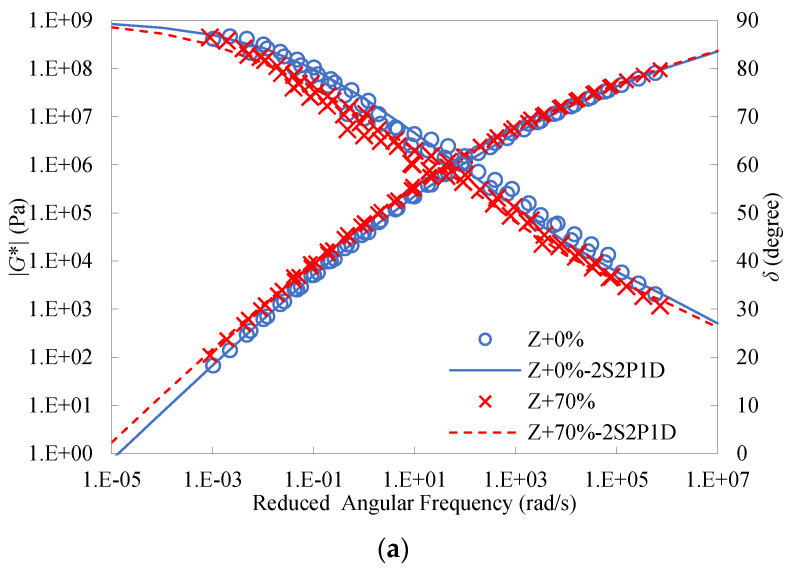

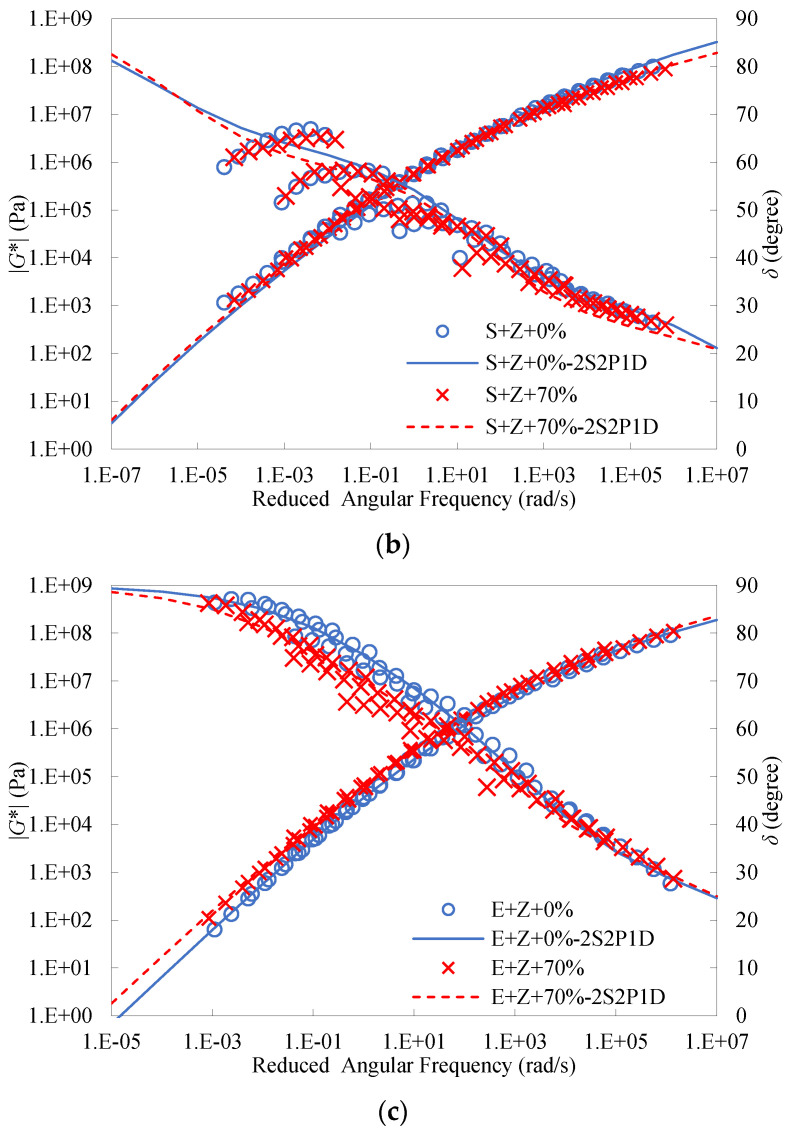
Calculated master curves of dynamic modulus and phase angle for: (**a**) Z binders; (**b**) S + Z binders; (**c**) E + Z binders.

In this study, the reference temperature *T*_r_ was selected as 28 °C for the construction of the master curves for the binders. Figure 16 presents the calculated master curves of dynamic modulus and phase angle for the three types of binders. As observed, the 2S2P1D model well simulated the dynamic modulus master curves for all the binders. For the Z and E + Z binders, the phase angle master curves of the 2S2P1D model were also in good agreement with the test data. However, due to the addition of S, the phase angle test data of the S + Z binders obtained at different test temperatures were incapable of forming single curves by horizontal shift, which led to the failure of the time-temperature superposition principle and unsatisfactory fitting of the 2S2P1D model. This may be because the WMA additive S is a thermorheologically complex material in essence. Table 14 summarizes the resulting parameters of the 2S2P1D model and fitting errors.

**Figure 17:**
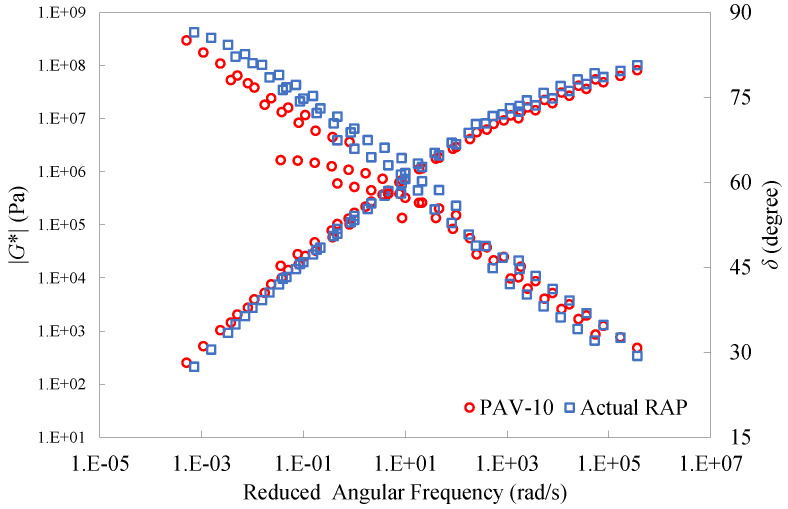
Master curves of dynamic modulus and phase angle for the actual RAP binder and the PAV-10 binder.

Figure 17 gives the master curves of dynamic modulus and phase angle for the actual RAP binder and the PAV-10 binder. As can be seen, the master curves of the two aged binders were very close to each other. This indicates that their linear viscoelastic properties were very similar, and the use of the PAV-10 binder as an alternative to the actual RAP binder was rational and effective.

**Figure 18:**
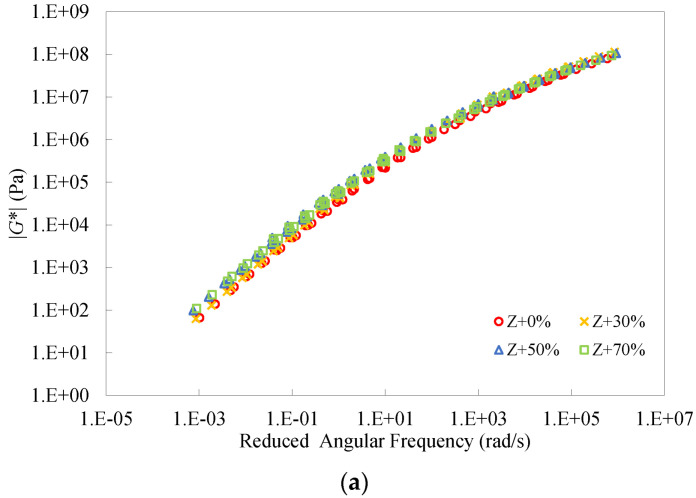

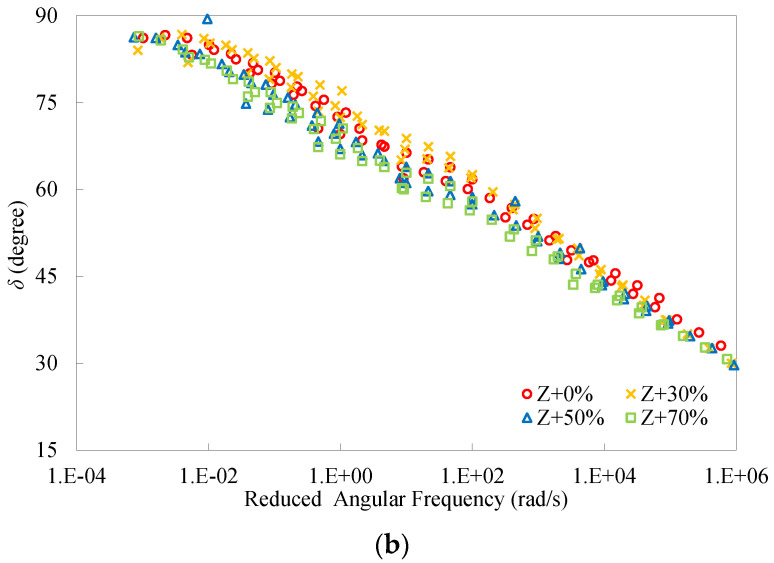
Master curves of dynamic modulus and phase angle of the Z binders at different RAP binder contents.

It can be seen from Figure 18 that both the master curves of dynamic modulus and phase angle at different RAP binder contents were very close for the Z binders. This further verifies the recycling effect of the rejuvenating agent. Even so, there still existed differences between the master curves, in particular when the percentage of the RAP binder became high. This also indicates that it is difficult for the rejuvenating agent to completely balance the negative impact of the aged binder.

**Figure 19:**
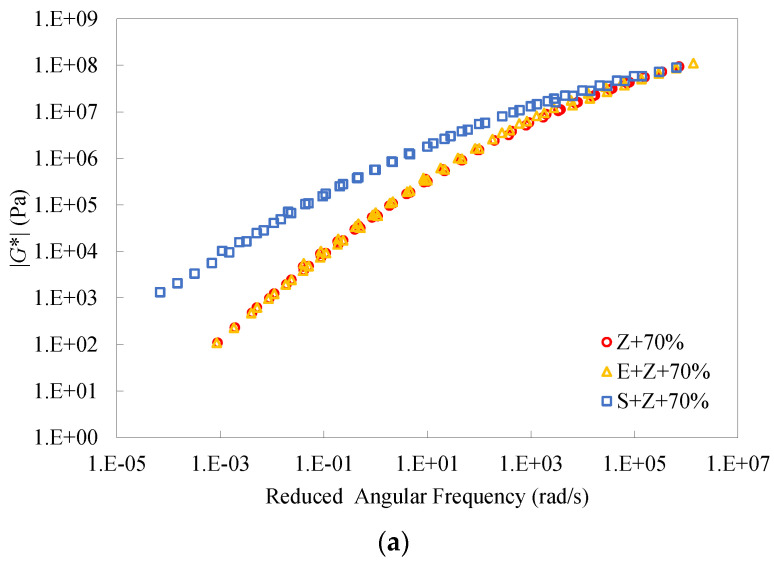

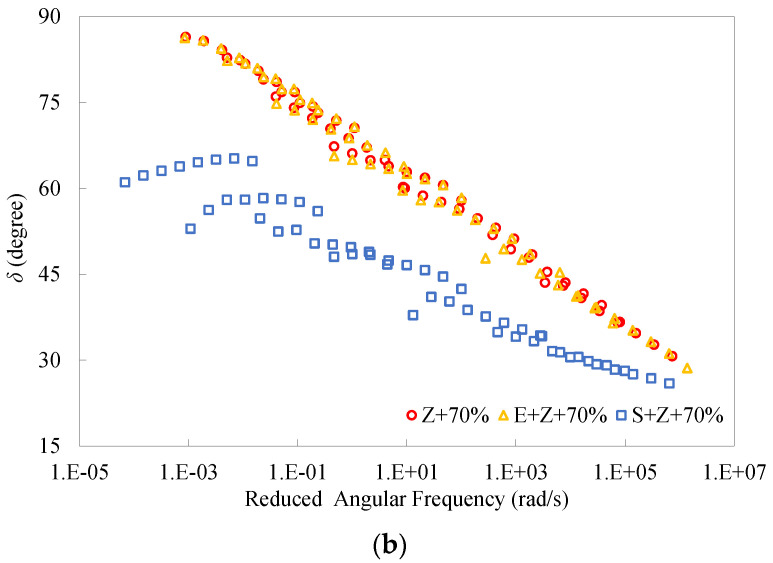
Master curves of dynamic modulus and phase angle of the warm-mix recycled binders at the RAP binder content of 70%.

Figure 19 shows the master curves of dynamic modulus and phase angle of the warm-mix recycled binders at the RAP binder content of 70%. It can be seen that the inclusion of S considerably improved the dynamic modulus and lowered the phase angle of the binders over a wide range of frequencies and temperatures, indicating the reinforcement effect on the stiffness and the enhancement effect on the elasticity. Moreover, the difference between the master curves of the S + Z binder and the other two binders became more pronounced at lower frequencies. Unlike the additive S, E had a very slight influence on the master curves of dynamic modulus and phase angle of the recycled binder at the RAP binder content of 70%. Similar observations can be attained for the binders at the other RAP binder contents, 0%, 30%, and 50%.

## 5. Conclusions

The present study investigated the rheological behavior of warm-mix recycled asphalt binders with high percentages of RAP binder. The combined impacts of two WMA additives (wax-based S and surfactant-based E), a rejuvenating aging (Z), four RAP binder contents (0~70%), and three aging states (unaged, short-term aged, and long-term aged) were assessed in detail using various rheological and performance tests over the entire range of service temperatures. On the basis of the results and analyses, the following conclusions can be drawn:

1. In terms of the conventional performance tests and the apparent viscosity test, the artificial RAP binder that was obtained by subjecting the virgin binder to successive RTFO aging and PAV aging with a specific exposure duration could achieve similar performance and aging degree to those of the actual one;
2. The rejuvenating agent Z could effectively alleviate the aging effect of the RAP binder; however, it was still difficult to completely eliminate this negative impact, in particular at higher RAP binder contents, despite a relatively accurate rejuvenating agent dosage by weight of the RAP binder (6%) used;
3. The addition of S remarkably lowered the apparent viscosity of the warm-mix recycled binders (by up to 35.0%), and with the decreasing temperature, this effect of viscosity reduction became more significant (within the range of 115 °C to 175 °C); however, the WMA additive E had little influence (with the reduction of only up to 5.2% in apparent viscosity) on the binder viscosity due to its surfactant nature;
4. Under both unaged and RTFO short-term aged conditions, the WMA additive S could considerably enhance the rutting performance (respectively with the increases of up to 399.5% and 411.3% in |G*|/sin*δ*) due to the wax crystallization in S, whereas E only enhanced the rutting resistance very slightly (respectively with the increases of up to 23.0% and 29.9% in |G*|/sin*δ*);
5. Both S and E were capable of improving the fatigue performance of the recycled binders, but E performed better than S in this aspect (respectively with the reductions of up to 42.3% and 22.1% in|G*|·sin*δ*);
6. The use of S had an adverse impact on the thermal cracking resistance (with an increase of up to 70.2% in *S*_a_ and a decrease of up to 34.1% in m-value between −24 °C and −12 °C), but the inclusion of E had a very mild influence on the low-temperature performance of the recycled binders;
7. The addition of S broadened the ranges of pavement service temperature by about two PG grades, 12 °C though S lowered the low-temperature performance of the binders (about one PG grade, 6 °C), whereas the use of E almost did not change the PG grades of the binders. Moreover, S could enhance the short-term aging resistance of the binders;
8. The inclusion of S considerably improved the dynamic modulus and lowered the phase angle of the binders over a wide range of frequencies and temperatures, indicating the reinforcement effect on the stiffness and the enhancement effect on the elasticity, but E had a very slight influence on the two master curves of the warm-mix recycled binders;
9. Due to the thermorheologically complex nature of S, the phase angle test data of the S + Z binders measured at different temperatures were incapable of forming single curves by horizontal shift, which led to the failure of the time-temperature superposition principle and unsatisfactory fitting of the 2S2P1D model.

This study served as a preliminary investigation of the rheological behavior of warm-mix recycled asphalt binders with high percentages of RAP binder. Additional research efforts are needed to evaluate the effects of other WMA techniques, like the foaming-based technology and other RAP sources. Besides, the relationships between the performance of the warm-mix recycled asphalt binders with high contents of RAP binder and the corresponding WMA-high RAP asphalt mixtures are recommended to develop. More advanced rheological tests, like the MSCR and LAS tests, are needed for the rheological evaluation of the materials in future studies.

## Figures and Tables

**Table 1:** Properties of the #90 and actual RAP binders.

Binder		Original				RTFO	PAV		
	Penetration at 25 °C (0.1 mm)	Softening Point (°C)	Ductility at 15 °C (mm)	Apparent Viscosity at 135 °C (Pa·s)	|G*|/sinδ (58 °C) (kPa)	|G*|/sinδ at 58 °C (kPa)	|G*|·sinδ at 25 °C (kPa)	*S*_a_ at −18 °C (MPa)	m-Value at −18 °C
#90	85.7	46.7	>1000	0.329	2.207	3.766	2278	260.5	0.324
Actual RAP	42.1	55.1	147.8	0.605	8.13	−	1456	288.5	0.325

**Table 2:** Basic properties of the rejuvenating agent ZGSB.

Saturates Fraction (%)	Aromatics Fraction (%)	Viscosity by Vacuum Capillary Viscometer at 60 °C (mm^2^/s)	Density at 15 °C (g/cm^3^)
23.9	59.9	153.5	0.987

**Table 3:** Percentage increase in apparent viscosity with the RAP binder content.

RAP Binder Content (%)	Percentage Increase (%)					
	115 °C			175 °C		
	Z	E + Z	S + Z	Z	E + Z	S + Z
0	0	0	0	0	0	0
30	13.5	13.2	31.1	59.7	39.7	55.4
50	25.7	25.4	53.9	68.1	51.3	73.8
70	35.4	**35.3**	**71.2**	79.2	**60.3**	**81.5**

**Table 4:** Percentage increase in apparent viscosity caused by the WMA additive.

Binder Type	Percentage Increase (%)							
	115 °C				175 °C			
	RAP-0%	RAP-30%	RAP-50%	RAP-70%	RAP-0%	RAP-30%	RAP-50%	RAP-70%
Z	0	0	0	0	0	0	0	0
E + Z	−0.4	−0.6	**−0.7**	−0.5	8.3	**−5.2**	−2.5	−3.1
S + Z	**−35.0**	−24.9	−20.4	−17.9	−9.7	**−12.1**	−6.6	−8.5

**Table 5:** Percentage increase in |G*|/sin*δ* with the RAP binder content under the unaged condition.

RAP Binder Content (%)	Percentage Increase (%)					
	46 °C			70 °C		
	Z	E + Z	S + Z	Z	E + Z	S + Z
0	0	0	0	0	0	0
30	16.8	27.2	15.5	8.1	25.2	14.3
50	59.4	46.1	39.8	53.4	37.7	37.9
70	82.1	63.7	53.0	**72.3**	**67.5**	**56.2**

**Table 6:** Percentage increase in |G*|/sin*δ* caused by the WMA additive under the unaged condition.

Binder Type	Percentage Increase (%)							
	46 °C				70 °C			
	RAP-0%	RAP-30%	RAP-50%	RAP-70%	RAP-0%	RAP-30%	RAP-50%	RAP-70%
Z	0	0	0	0	0	0	0	0
E + Z	10.8	**20.6**	1.5	−0.4	6.2	**23.0**	−4.7	3.3
S + Z	**399.5**	393.9	338.3	319.7	317.0	**340.8**	274.8	278.0

**Table 7:** Percentage increase in |G*|/sin*δ* with the RAP binder content under the short-term aged condition.

RAP Binder Content (%)	Percentage Increase (%)					
	46 °C			70 °C		
	Z	E + Z	S + Z	Z	E + Z	S + Z
0	0	0	0	0	0	0
30	8.1	16.4	6.5	20.8	24.9	18.5
50	25.9	32.5	10.6	38.9	42.6	22.9
70	46.6	52.5	17.2	68.8	71.6	24.5

**Table 8:** Percentage increase in |G*|/sin*δ* caused by the WMA additive under the short-term aged condition.

Binder Type	Percentage Increase (%)							
	46 °C				70 °C			
	RAP-0%	RAP-30%	RAP-50%	RAP-70%	RAP-0%	RAP-30%	RAP-50%	RAP-70%
Z	0	0	0	0	0	0	0	0
E + Z	20.6	**29.9**	26.8	25.4	21.9	**26.0**	25.2	23.9
S + Z	**411.3**	403.8	349.1	308.9	**335.3**	327.1	285.2	221.0

**Table 9:** Percentage increase in |G*|·sin*δ* with the RAP binder content under the long-term aged condition.

RAP Binder Content (%)	Percentage Increase (%)					
	13 °C			22 °C		
	Z	E + Z	S + Z	Z	E + Z	S + Z
0	0	0	0	0	0	0
30	8.2	11.7	14.2	8.8	15.5	9.1
50	13.5	24.9	26.7	10.4	26.8	15.5
70	23.4	33.8	37.0	19.0	41.1	30.1

**Table 10:** Percentage increase in |G*|·sin*δ* caused by the WMA additive under the long-term aged condition.

Binder Type	Percentage Increase (%)							
	13 °C				22 °C			
	RAP-0%	RAP-30%	RAP-50%	RAP-70%	RAP-0%	RAP-30%	RAP-50%	RAP-70%
Z	0	0	0	0	0	0	0	0
E + Z	−36.9	**−39.3**	−36.2	−32.4	**−42.3**	−38.8	−33.8	−31.7
S + Z	**−22.1**	−20.0	−16.9	−13.9	**−12.1**	−11.9	−8.0	−3.9

**Table 11:** Percentage increases of *S*_a_ and m-value with the RAP binder content under the long-term aged condition.

Quantity	RAP Binder Content (%)	Percentage Increase (%)								
		−24 °C			−18 °C			−12 °C		
		Z	E + Z	S + Z	Z	E + Z	S + Z	Z	E + Z	S + Z
*S*_a_	0	0	0	0	0	0	0	0	0	0
	30	−14.1	−12	−10.2	−13.4	−16.6	−7.9	−27.9	−44.2	−17.4
	50	−30.1	−24.5	−13.2	−29.6	−20.6	−11	−34.5	−36.3	−6.2
	70	−21.9	−21.2	−3.7	−22.6	−23.1	−2	−13.1	−17.9	−4.3
m-value	0	0	0	0	0	0	0	0	0	0
	30	−7.6	7.5	1.8	−8.4	3.1	5.1	−12.5	10.7	7.8
	50	12	23	5.5	−7.5	5.2	3.3	−19.9	−6.8	1.5
	70	−12.6	12.4	2.3	−0.8	2.7	2.4	−21.8	5	2.5

**Table 12:** Percentage increases of *S*_a_ and m-value caused by the WMA additive under the long-term aged condition.

Quantity	Binder Type	Percentage Increase (%)											
		−24 °C				−18 °C				−12 °C			
		RAP-0%	RAP-30%	RAP-50%	RAP-70%	RAP-0%	RAP-30%	RAP-50%	RAP-70%	RAP-0%	RAP-30%	RAP-50%	RAP-70%
*S*_a_	Z	0	0	0	0	0	0	0	0	0	0	0	0
	E + Z	−0.8	1.7	7.1	0.1	0.6	−3.1	13.4	0	9.7	−15.1	6.7	3.6
	S + Z	4.2	8.9	**29.3**	28.5	−5.6	0.4	19.3	**19.6**	18.9	36.2	**70.2**	31
m-value	Z	0	0	0	0	0	0	0	0	0	0	0	0
	E + Z	−13.9	0.2	−5.4	10.7	−2.6	9.7	10.8	0.8	−15.3	7.1	−1.5	13.8
	S + Z	−7	2.5	**−12.4**	8.7	**−16.2**	−3.8	−6.5	−13.6	**−34.1**	−18.9	−16.6	−13.6

**Table 13:** Performance grading (PG) grades of the warm-mix recycled binders.

Binder	PG High (Original)	PG High (RFTO)	PG Intermediate (RTFO + PAV)	PG Low (RTFO + PAV)	PG Grade
S + Z + 0%	76	70	16	−12	70–22
S + Z + 30%	76	76	19	−12	76–22
S + Z + 50%	76	76	19	−12	76–22
S + Z + 70%	76	76	19	−12	76–22
E + Z + 0%	64	58	16	−18	58–28
E + Z + 30%	64	58	16	−18	58–28
E + Z + 50%	64	58	16	−18	58–28
E + Z + 70%	64	58	19	−18	58–28
Z + 0%	64	58	19	−18	58–28
Z + 30%	64	58	19	−12	58–22
Z + 50%	64	58	19	−18	58–28
Z + 70%	64	58	22	−18	58–28

**Table 14:** Resulting parameters of the 2S2P1D model and fitting errors for the binders.

Sample	*α*	*k*	*h*	*β*	*τ*_0_ (s)	*f* (%)
Actual RAP	8.22	0.33	0.68	9.20 × 10^1^	4.18 × 10^−6^	1.92
Pav-10	4.46	0.21	0.56	2.12 × 10^3^	2.28 × 10^−7^	3.68
Z + 0%	7.50	0.36	0.69	8.37 × 10^1^	8.98 × 10^−7^	1.37
Z + 30%	6.78	0.35	0.70	4.87 × 10^1^	1.75 × 10^−6^	1.77
Z + 50%	8.87	0.37	0.71	6.58 × 10^1^	2.63 × 10^−6^	1.63
Z + 70%	9.21	0.36	0.70	1.01 × 10^2^	1.75 × 10^−6^	1.01
E + Z + 0%	8.69	0.32	0.69	6.97 × 10^1^	9.76 × 10^−7^	1.31
E + Z + 30%	9.47	0.33	0.69	9.36 × 10^1^	9.31 × 10^−7^	1.46
E + Z + 50%	9.36	0.35	0.70	8.70 × 10^1^	1.75 × 10^−6^	1.39
E + Z + 70%	8.93	0.34	0.69	1.08 × 10^2^	1.75 × 10^−6^	1.70
S + Z + 0%	14.19	0.34	0.69	1.84 × 10^3^	2.68 × 10^−5^	4.00
S + Z + 30%	14.16	0.31	0.67	3.22 × 10^3^	2.22 × 10^−5^	3.78
S + Z + 50%	23.46	0.31	0.70	8.36 × 10^2^	3.86 × 10^−5^	3.21
S + Z + 70%	16.19	0.28	0.65	4.39 × 10^3^	1.18 × 10^−5^	3.24

## Data Availability

Data sharing is not applicable to this article.
